# Neurosurgical management of brain metastases in the elderly: a prospective study on adverse event prevalence and predictors

**DOI:** 10.1007/s10143-025-03338-y

**Published:** 2025-02-15

**Authors:** Pavlina Lenga, Moritz Scherer, Helena Kleineidam, Andreas Unterberg, Sandro M. Krieg, Philip Dao Trong

**Affiliations:** 1 Department of Neurosurgery, Heidelberg University Hospital, University of Heidelberg, Im Neuenheimer Feld 400, Heidelberg, 69120 Germany; 2 Medical Faculty of Heidelberg University, Heidelberg, Germany

**Keywords:** Adverse events, Brain metastases, Older age, Clavien-Dindo classification system

## Abstract

The management of brain metastases (BM) in geriatric patients poses significant challenges in the context of an aging population and advances in systemic cancer treatment. This study provides insights into the prevalence and nature of adverse events (AEs) following intracranial surgery in patients aged 65 years and older. It highlights the complexities and implications of treating this demographic patient population and identifies risk factors associated with AEs. This prospective study includes patients aged 65 years and older with BM who underwent surgery between January 2022 and December 2023. A detailed assessment of AEs, defined as any complication occurring within the first 30 days post-surgery, was conducted. Potential risk factors for the occurrence of AEs were examined. The study encompassed 104 patients, averaging 70.1 ± 2.8 years, with 102 undergoing surgery. The mean age-adjusted Charlson Comorbidity Index (CCI) score was 8.9 ± 1.2, indicating a significant comorbidity burden, predominantly cardiac conditions. The Karnofsky Performance Scale (KPS) showed substantial improvement post-surgery, increasing from 71.3% ± 7.8 to 75.1% ± 5.0 (*p* = 0.045). The average hospital stay was 10.6 days. Four non-surgery-related mortalities occurred within the 30-day postoperative period. Surgery-related AEs included wound complications in two patients, with one necessitating surgical revision. Advanced age and comorbidities emerged as significant predictors of AEs. Our findings suggest that neurosurgical intervention for BM in the elderly is a feasible and safe option, demonstrating favorable morbidity and mortality rates. However, careful postoperative monitoring is crucial, especially considering the baseline health status of these patients, which increases their susceptibility to AEs. Standardizing protocols for AE reporting and analysis is essential for improving clinical outcomes and maintaining the quality of healthcare for this patient population.

## Introduction

In industrialized nations, advancements in healthcare delivery systems have notably increased life expectancy, culminating in a greater proportion of the elderly population. This demographic shift has concomitantly led to a surge in the incidence and mortality rates of cancer among older individuals [1–3]. Brain metastasis (BM) is the most common neurologic complication of systemic malignancies, manifesting in 10–30% of cancer patients [3, 4]. The escalating number of elderly patients necessitates a reassessment of the most efficacious therapeutic strategies for BM [5–7]. The correlation between advanced age and increased morbidity underscores the complexity of decision-making in this demographic population. While there has been a noted decrease in overall cancer mortality, the incidence of brain metastases has been increasingly diagnosed in clinical settings. Contemporary standards of care dictate a multimodal treatment paradigm, typically initiating with surgical resection of the intracranial neoplasm [8]. Class I evidence is available that demonstrates that surgical resection followed by radiotherapy (RT) leads to significantly lower rates of local recurrence when compared with RT alone (Patchel et al.). It is imperative to underscore that such surgical interventions aim for maximal safe resection while minimizing neurological deficits ideally resulting in a prolongation of survival while prioritizing the preservation or improvement of the patient’s quality of life [9]. However, the potential for surgery-related adverse events (AEs) remains a significant concern, as these may result in new neurological deficits, diminished quality of life, or even mortality. This is particularly pertinent in the elderly, where baseline health status may be compromised, increasing their susceptibility to postoperative AEs [10, 11]. There exists an urgent need for precise, objective preoperative risk stratification tools to assist surgeons in evaluating the risk-to-benefit ratio and facilitate informed clinical decisions. Hence, a comprehensive preoperative evaluation should integrate the assessment of life expectancy, quality of life, and potential predictors of surgical outcomes to inform optimal management strategies. While extensive research has been conducted on morbidity and mortality in the context of cardiothoracic and general surgeries among older adults, there is a paucity of data regarding optimal therapeutic approaches for elderly patients undergoing neurosurgical interventions for BM. This gap in knowledge extends to the identification of potential AEs and confounding factors that may influence their incidence [12–14].

In response to this knowledge deficit, we have initiated the compilation of a prospectively assembled database with a focus on the surgical management of BM in patients aged 65 years and older. Our objective is to delineate their clinical trajectories, analyze morbidity and mortality outcomes, and discern potential risk factors that may predispose them to AEs.

## Methods

In the present investigation, we employed a prospective methodology, collecting data from a single-center neurosurgical tertiary care institution. Ethical approval for the study was granted by our institutional ethics committee, under the reference number S-425/2022, in accordance with the ethical principles of the Declaration of Helsinki. The requirement for individual informed consent was deemed unnecessary as the completion of Post-Operative Adverse Event (POPAE) forms is an integrated component of our institutional protocol, which conforms with routine clinical practice. Our procedural adherence to data acquisition and analytical methods is consistent with those set forth in our preceding publications [15, 16]. This entailed a rigorous comparative analysis of our specialized POPAE database against the aggregate administrative hospital data. Quality assurance was reinforced through systematic quarterly reviews, allowing for the rectification of any data inconsistencies, thereby fortifying the robustness of our dataset.

The POPAE database is scrupulously curated and contemporaneously updated by a dedicated team comprising 15 board-certified neurosurgeons and 18 neurosurgical trainees. Each patient, at the point of discharge, was furnished with a POPAE form, meticulously completed by the responsible physician of the respective ward. Prior to database entry, a senior consultant performed a validation of the data. Readmissions within a 30-day window postoperatively triggered an immediate notification to the medical team. Case complexities were collaboratively reviewed at recurring Morbidity and Mortality Conferences (MMCs), ensuring a collective and iterative approach to clinical analysis within the neurosurgical department. The analytic focus of this study was on patients aged 65 years and older suffering from an intracranial BM.

The terminologies and methodological criteria central to this study have been comprehensively expounded in our prior research outputs [15, 16]. This establishes a uniform application of terminologies and analytical criteria across our research endeavors, facilitating comparability and methodological coherence. *‘Surgical goal not achieved’ refers to cases in which intended surgical objectives*,* such as complete resection of the dominant lesion or adequate decompression*,* were not met. This classification follows the criteria defined in our previous study* [15].*”*

In addition to the prospective methodology, we collected comprehensive data on key patient and surgical factors. These include patient demographics (age, sex, comorbidities), clinical presentation (neurological deficits, imaging findings), surgical details (indication for surgery, extent of resection), and postoperative outcomes (incidence of adverse events, length of hospital stay, and 30-day mortality). Our data collection process aligns with the standardized methodology previously described by Lenga et al. [16–18] and Dao Trong et al. (2022), which detail our criteria for adverse event classification and postoperative outcome measurement. Preoperative health conditions were evaluated utilizing the age-adjusted Charlson Comorbidity Index (CCI), with each patient receiving a score reflective of their comorbidity status, stratified into categories of none, minimal, moderate, or severe (CCI scores of 0, 1–2, 3–5, and over 5, respectively) [19, 20]. Data on patient mobility and self-care capacity post-intervention were garnered from the most recent medical assessments available. Functional status and independence were gauged using the Karnofsky Performance Index (KPI), which assigns functional grades from a full independence score of 100 to complete dependency or death, scored at 0, with interim scores reflecting varying levels of required assistance and capability for daily activities [21].

### Procedures

In this study, each patient’s treatment plan was discussed in a multidisciplinary tumor board comprising neurosurgeons, medical oncologists, radiation oncologists, and palliative care specialists. The board meetings were held weekly, during which patient-specific factors—including tumor location, comorbidities, performance status, and individual goals of care—were reviewed to determine the optimal management approach. For patients undergoing surgery, the decision was made based on consensus regarding the anticipated benefits of resection for symptomatic relief or prolongation of quality-adjusted life expectancy.

For patients with multiple brain metastases, the number of lesions was recorded. Surgical intervention was primarily indicated for patients with a dominant lesion causing significant mass effect, symptomatic edema, or requiring histological diagnosis. Mass effect was defined as midline shift ≥ 5 mm, uncal herniation, or ventriculomegaly secondary to compression of cerebrospinal fluid pathways. The decision for surgery in cases of multiple metastases was guided by the presence of a single symptomatic lesion that warranted immediate intervention.

Postoperatively, the rehabilitation needs of each patient were assessed in collaboration with physical therapists and rehabilitation medicine specialists. Rehabilitation strategies were tailored based on the patient’s functional status (as determined by KPS) and comorbid conditions, with an emphasis on early mobilization, physical therapy, and occupational therapy to improve functional recovery and quality of life. This multidisciplinary approach ensured that each patient received individualized and comprehensive care throughout the treatment continuum.

### Statistical analysis

Categorical variables were enumerated and represented as proportions. Continuous variables, upon confirmation of normal distribution via the Shapiro-Wilk test, were denoted as means with standard deviations. Baseline demographics and surgical parameters were subjected to univariate analysis for comparative purposes. Subsequently, a binary logistic regression model was employed to elucidate potential risk factors associated with postoperative adverse events. A *p*-value of less than 0.05 was predetermined as the cut-off for statistical significance. All statistical computations were conducted using the statistical software package SPSS, version 24.0.0.0 (IBM Corp., Armonk, NY, USA).

## Results

### Baseline characteristics

Out of 204 consecutively screened brain metastases patients from January 2022 to December 2023, 104 patients were 65 years and older (70.1 ± 2.8 years) and underwent surgical intervention. 50.0% of the cohort (52/104) presented with multiple intracranial metastases. Of these, a substantial 84.6% (88 out of 104) represented elective surgeries, while the remaining 15.4% (16 out of 104) were emergency procedures. Only two patients were treated with conservative management strategies. The most common surgical site was the frontal lobe, accounting for 36.5% (38 out of 104) of the interventions. Lung cancer emerged as the most frequently encountered carcinoma, with a prevalence of 60.8% (62 out of 102). Utilizing the age-adjusted Charlson Comorbidity Index (CCI) to evaluate comorbidity burden, the mean score was determined to be 8.9 ± 1.2, with cardiac conditions being the most prevalent comorbidity in the cohort. A detailed demographic breakdown of the study’s population is provided in Table 1.

**Table 1:** Baseline characteristics

	*N* = 104	%
Age, years (mean, SD, range)	70.1 (2.8; 65–91)	
Sex (n, %)		
Male	53	51.0
Female	51	49.0
Non-Elective	14	13.5
Elective	88	84.6
Conservative	2	1.9
Comorbidities		
Age-adjusted CCI score (mean, SD)	8.9 (1.2)	
Arterial hypertension	67	64.4
Myocardial infarction	22	21.2
Coronary heart disease	23	22.1
Atrial fibrillation	55	52.9
Heart failure	12	11.5
Peripheral vascular disease	4	3.8
COPD	22	21.2
Diabetes mellitus Type II	55	52.9
Renal failure	28	26.9
Liver disease	5	4.8
Gastrointestinal ulcer	5	4.8
TIA/stroke	19	18.3
Malignancy	104	100.0
Dementia	12	11.4
Alcohol abuse	8	7.7
Location		
Frontal	38	36.5
Parietal	8	7.7
Temporal	15	14.4
Occipital	8	7.7
Cerebellum	27	26.0

SD, standard deviation

**Table 2:** Peri- and postoperative surgical characteristics and clinical course across 102 patients undergoing surgery

	*n* = 102
Surgical duration, min	287.5 (111.2)
KPI preOP (median, IQR)	70.0 (10.0)
KPI postOP (median, IQR)	75.0 (10.0)
Hospital stay, days	10.6 (5.1)
Mortality (n, %)	4 (3.9)
Histology (n, %)	
Lung	62 (60.8)
Breast	20 (19.6)
Colorectal	3 (2.9)
Melanoma	10 (9.8)
Kidney	3 (2.9)
Prostata	4 (3.9)
Pancreas	1 (1.0)
Multiple Myeloma	1 (1.0)

Except where otherwise indicated, quantities are mean (SD)

Furthermore, we employed the Diagnosis-Related Graded Prognostic Assessment (GPA) to estimate the median survival of our patient cohort. Notably, the mean GPA was 2.2 ± 0.6, indicating a median survival of 8.4 months ± 3.0. 84.6% of the patients (88 out of 104) received postoperative radiation, while 26.9% (28 out of 104) underwent targeted chemotherapy [22].

### Surgical characteristics and occurrence of AEs

Within our study’s framework, 102 patients underwent surgical intervention. Of the 102 patients who underwent surgery, 28 patients (27.5%) presented with multiple brain metastases. The median number of metastases in these patients was 3 (range: 2–5). In all cases of multiple lesions, surgical intervention targeted the dominant metastasis, which was responsible for significant mass effect or symptomatic progression. Among these 28 patients, 82% (23/28) exhibited clinical signs of mass effect, such as midline shift (≥ 5 mm) or hydrocephalus requiring urgent decompression. The remaining 18% (5/28) underwent surgery for histological confirmation to guide systemic therapy in the context of diagnostic uncertainty. The peri/and postoperative clincal course is depeicted in Table 2.

The preoperative functional status of patients, as gauged by the KPS, showed marked improvement postoperatively, rising from an average of 71.3% ± 7.8 to 75.1% ± 5.0 (*p* = 0.045). The average length of hospital stay recorded was 10.6 days ± 5.1. Within the 30-day follow-up period after surgery, there were four mortalities. The causes of death were attributed to severe pulmonary embolism and pneumonia leading to respiratory insufficiency, affecting two patients in each category respectively. Regarding surgery-related adverse events (AEs), there were incidents of wound complications in two patients, with one requiring surgical revision. Dural leaks were observed in only three cases; however, all necessitated surgical intervention for correction. Eight patients developed new neurological deficits post-surgery. Among these, four patients had CDC Grade 1 deficits, including one with hemiparesis and another with sensory aphasia. Additionally, four patients exhibited Grade 2 deficits, characterized by a significant decrease in Glasgow Coma Scale (GCS) scores to 12, due to increased postoperative edema as observed in CT scans. However, none required additional surgical treatment. A comprehensive account of these outcomes is detailed in Table 3. Table 4 elaborates on non-surgery related AEs, with urinary tract infections as the most common, succeeded by pulmonary embolism, pneumonia, acute renal failure, and respiratory insufficiency, all sharing a similar prevalence rate.

**Table 3:** Summary of surgery-related adverse events

	*n*	%	Revision surgery	%
Wound event	2	2.0	1	1.0
Dural leak	3	2.9	3	2.9
Postoperative infection	0	0.0	0	0.0
New neurological deficits	8	7.8	0	0.0
Rebleeding	0	0.0	0	0.0
Surgical goal not achieved	0	0.0	0	0.0
Secondary transfer to IMC or ICU	6	5.9	0	0.0

IMC, intermediate care unit; ICU, intensive care unit

**Table 4:** Summary of not surgery-related adverse events

	*n*	%
Acute renal failure	2	1.9
Respiratory deficiency	2	1.9
Heart failure	1	1.0
Pneumonia	2	1.9
Pulmonary embolism	2	1.9
Urinary tract infection	3	2.9

### Risk factors for AEs

*The binary logistic regression analysis revealed that the coexistence of comorbid conditions (OR 1.8*,* 95% CI 1.1–5.2) and advanced age (OR 1.9*,* 95% CI 1.1–3.2) were statistically significant predictors for the incidence of adverse events*,* as shown in* Table 5.

**Table 5:** Risk factors associated with surgery related AEs

Risk factor	OR (95% CI)	*p*-value
Age-adjusted CCI score	1.8 (1.1–5.2)	**0.045**
Age	1.9 (1.1–3.2)	**0.004**
KPI	1.2 (0.9–1.2)	0.781
Duration of surgery	1.4 (1.1–1.9)	0.076
Hospital stay	0.5 (0.1–1.5)	0.778

CCI, Charlson Comorbidity Index; CI, confidence interval; OR, odds ratio

## Discussion

The increasing focus on differentiating geriatric neurosurgery from general neurosurgery becomes paramount when considering patients with underlying malignancies. This distinction is critical in risk assessment and management of these distinct patient populations, potentially being a key factor in achieving positive outcomes and providing optimal, patient-centered care [23]. It is important to note that over 25% of patients with brain metastases (BM) are elderly [12, 24–26], underlining the urgent need to prioritize older patients. Enhancing preoperative assessment, customizing surgical goals, and improving postoperative care are essential steps towards benefiting this demographic.

Our study, to the best of our knowledge, is the first to explore the prevalence of adverse events and the 30-day mortality rates. In our consecutive series, every second BM patient was over 65 years old. This exploration is based on a large, prospectively compiled AE database from our tertiary center. Our findings indicate that surgery-related AEs occurred in 12.7% of cases, while non-surgical complications were present in 11.8%. Notably, the patients had a high average CCI of 8.9 points, indicating a challenging baseline health status. Despite this, the functional Karnofsky status before surgery was relatively good, with an average score of 70.0%, which notably improved post-surgery. It is critical to highlight that the 30-day postoperative mortality rate was relatively high at 3.9%, attributed not to surgical factors but to medical complications like cardiac and respiratory issues. Our study adds evidence that significant predictors for the occurrence of AEs included the patients’ increasing age and a higher rate of comorbidities.

In a recent study by Zhang et al. (2021), an investigation into 30-day postoperative systemic complications was conducted on 212 geriatric patients undergoing surgery for various intracranial tumors [14]. The findings of this study indicated a 24.5% incidence of systemic complications, a statistic that is consistent. However, it is crucial to highlight that Zhang et al.‘s study did not exclusively focus on complications directly related to surgery. Notably, systemic infections were the most common complication in their study, with a prevalence of nearly 14%. Tomita and Raimondi (1981) conducted a retrospective analysis on 80 elderly patients with brain tumors. They discovered that 28.8% of these patients experienced postoperative systemic complications, with pulmonary issues being the most frequent [27]. Echoing these findings, Asano et al. (2009) reported systemic complications in approximately 40.0% of their study cohort [28]. A significant limitation of these aforementioned studies is their failure to clearly differentiate between complications that are directly attributable to surgery and those that are not. This lack of distinction obscures the true impact of surgical intervention on such a vulnerable patient group. In contrast, Ferroli et al. (2021) reported neurosurgical complications in 46% of their study cohort of 143 older patients with various tumor types. Interestingly, 33.6% of these complications did not necessitate invasive treatment, thus only 12.4% of patients required such interventions [29]. These findings bear resemblance to those observed in our study but exhibit a higher incidence rate. The primary distinction between the aforementioned studies and ours lies in our specific focus on elderly patients with BM. This specific focus potentially limits the direct comparability with studies involving other types of brain tumors, such as meningiomas or gliomas, which might involve longer surgeries and higher risks, such as increased blood loss, hence potentially leading to a higher rate of postoperative systemic complications. It is therefore important to stress that BM surgery may bear fewer risks of AEs than other cranial pathologies in elderly patients. Our study uniquely segregates surgical and non-surgical related complications, aiming to provide a more accurate portrayal of potential AEs in elderly patients following surgery.

In this study, we demonstrated that both increasing age and comorbidity status are significant predictors of AEs. This finding aligns with Zhang et al.‘s retrospective analysis of 212 patients with intracranial pathologies, which highlighted a significant association between comorbidities and postoperative complications [14]. Similarly, Asano et al. identified systemic complications as being influenced by factors such as a lower KPS, increased intraoperative blood loss, and variations in hemoglobin levels [28]. Notably, these studies did not identify chronological age as a significant factor in the occurrence of AEs. In contrast, our findings resonate with previous evidence., who reported that advanced age, particularly over 80 years, along with a higher CCI, are significant predictors of poorer outcomes, including AEs [30]. The heightened risk in geriatric surgical management primarily stems from two factors: the prevalence of medical comorbidities in the elderly and the functional decline associated with aging. The concept of frailty encapsulates this decline, characterized by a diminished physiological reserve and heightened vulnerability to stressors due to cumulative declines across multiple organ systems, which consequently leads to a higher incidence of adverse outcomes. Given these risks, comprehensive preoperative screening is crucial to predict outcomes accurately. Overall, this study underscores the complexity of managing geriatric patients in surgical contexts, highlighting the need for careful assessment and personalized approaches to optimize outcomes.

The management of brain metastases requires a multi-faceted approach, especially when considering long-term outcomes such as local recurrence and overall survival. Surgery serves as a critical intervention for patients with symptomatic lesions causing mass effect or elevated intracranial pressure. However, surgical resection alone is insufficient in most cases, as microscopic tumor cells frequently remain beyond the margins of resected tissue. This necessitates the integration of postoperative radiotherapy to optimize disease control. Radiosurgery, particularly SRS, has been increasingly favored following resection of brain metastases due to its ability to provide precise, high-dose radiation to the tumor bed, minimizing exposure to surrounding healthy brain tissue [31, 32]. Evidence has consistently demonstrated that SRS following resection offers superior local control compared to observation alone while avoiding the neurocognitive decline often associated with WBRT [31]. Brown et al. showed that adding SRS to the surgical cavity significantly reduced local recurrence without the substantial cognitive side effects seen with WBRT, positioning it as a standard of care for patients with limited brain metastases who undergo resection. WBRT, although effective in reducing the risk of both local and distant recurrence, has been associated with a higher risk of neurotoxicity, particularly in the elderly population [33]. For instance, Chang et al. demonstrated that patients treated with WBRT exhibited significantly more cognitive decline compared to those treated with SRS alone. As a result, WBRT has been gradually deprioritized in favor of more focal approaches like SRS, especially in patients with a limited number of metastases. Nevertheless, in cases of multiple metastases or high-risk patients, WBRT remains a valid therapeutic tool, providing comprehensive coverage to the entire brain and reducing the risk of distant metastatic spread [34]. The decision between SRS and WBRT must be individualized based on patient factors, such as age, functional status, and the number of metastases, to balance between disease control and quality of life. Moreover, several studies have investigated the combination of surgery and postoperative radiotherapy and its impact on survival. Patchell et al. demonstrated that the addition of WBRT following surgical resection significantly reduced both local and distant recurrence compared to surgery alone, underscoring the importance of adjuvant radiotherapy for improving disease-free intervals [35]. More recent work has focused on optimizing radiotherapy techniques to preserve cognitive function while maintaining oncological outcomes. For instance, Gondi et al. showed that hippocampal-sparing WBRT, combined with memantine, can help mitigate cognitive decline, thereby making WBRT a more viable option for selected patients Incorporating SRS or WBRT after surgery addresses residual microscopic disease, providing a survival advantage and significantly reducing the risk of local failure [36]. In our study cohort, the use of adjuvant radiotherapy was critical in achieving optimal outcomes, and we acknowledge that the combination of these treatment modalities is what ultimately provides the best potential for prolonged survival and improved quality of life [37].

Our study provides an in-depth, prospective evaluation of elderly patients undergoing surgical resection for BM, emphasizing the complex interplay between age, frailty, and comorbidity in determining perioperative outcomes. Previous studies have laid a strong foundation regarding the impact of frailty on surgical outcomes in BM patients, notably Kazemi et al. and Lim et al. who demonstrated the predictive value of frailty and comorbidity indexes, such as the Hospital Frailty Risk Score (HFRS) and sarcopenia measures [38, 39]. In our cohort, 50% presented with multiple metastases, and the high CCI prevalent. Our prospective data extends the findings of previous works by providing an ongoing, real-time assessment of how frailty and comorbidities interact with surgical outcomes. Unlike previous retrospective analyses, our approach allows us to precisely assess these parameters and how they directly correlate with adverse outcomes. Binary logistic regression identified both a high comorbidity burden (OR 1.8, 95% CI 1.1–5.2) and advanced age (OR 1.9, 95% CI 1.1–3.2) as independent predictors of adverse events, consistent with findings from other surgical cohorts, such as those reported by Lucido et al. [40]. These results validate the significance of frailty metrics as robust predictors of surgical risk and support the growing emphasis on comprehensive preoperative assessments for BM patients.

Importantly, our study also highlights the functional benefits achievable in appropriately selected patients, as demonstrated by the significant improvement in Karnofsky Performance Status (KPS), from 71.3% ± 7.8 preoperatively to 75.1% ± 5.0 postoperatively (*p* = 0.045). This result indicates that surgery, when performed in carefully selected older patients, can improve functional outcomes and quality of life. This finding aligns with the conclusions of Lucido et al., who emphasized that frail patients could still benefit from intervention, provided that they are appropriately managed. Regarding postoperative interventions, the high rate of adjuvant radiation (84.6%) observed in our cohort underscores the importance of local control following resection [40]. As indicated by Brown et al., postoperative SRS has emerged as a crucial adjunct to surgery for enhancing local control while avoiding the neurocognitive risks associated with WBRT [31]. However, despite aggressive management strategies, the observed median survival of 8.4 ± 3.0 months in our cohort highlights the challenging prognosis of these patients, particularly those with multiple metastases and high comorbidity burdens. The postoperative AE profile in our cohort was also notable. Surgical complications included wound issues and dural leaks, while non-surgical AEs were predominantly infections, such as urinary tract infections and pneumonia. These complications align with findings by Colasacco et al., who reported that frail elderly patients with BM are at a high risk of developing postoperative infections and thromboembolic events [41]. Our real-time data collection provides a clear understanding of the burden of AEs in elderly surgical patients and highlights areas where intervention may be possible to mitigate risk. Given these findings, there is a critical need for tailored Early Recovery After Surgery (ERAS) protocols to address the specific vulnerabilities of elderly patients with brain metastases. The prospective data from our study provide an invaluable opportunity to develop and implement ERAS pathways designed to mitigate risk factors such as frailty, comorbidities, and infection susceptibility. Key components of such an ERAS approach could include preoperative nutritional optimization, targeted physical rehabilitation to address sarcopenia, standardized intraoperative protocols to reduce anesthesia-related complications, and rigorous postoperative monitoring to promptly identify and treat infections and thromboembolic events. Kazemi et al. have highlighted the positive impact of individualized perioperative care on outcomes for frail patients, and our findings support the extension of these principles to BM patients undergoing surgery [38]. Implementation of a tailored ERAS protocol, informed by our data, has the potential to reduce adverse event rates, enhance functional recovery, and ultimately improve the overall prognosis of elderly BM patients. Specifically, the integration of frailty assessments into perioperative care planning will be crucial to achieving these outcomes, allowing for risk stratification and personalized interventions that target modifiable factors. In conclusion, while prior literature has demonstrated the impact of frailty and comorbidity on outcomes in brain metastasis management, our prospective study adds an important dimension by capturing detailed perioperative and outcome data in real time. This approach allows for an improved understanding of the predictive factors for adverse outcomes and emphasizes the value of surgery in carefully selected patients despite significant frailty. Our findings strongly advocate for the development and integration of ERAS protocols specifically tailored to mitigate the risks associated with frailty and comorbidities in this vulnerable population, thereby improving the overall quality of care and long-term outcomes.

The protracted average hospitalization duration of 10.6 days observed in older patients undergoing cerebral surgery for metastasis warrants a detailed examination of non-surgical complications as primary contributors. Postoperative pneumonia is particularly detrimental in geriatric patients due to their reduced physiological reserves and the prevalence of comorbidities. According to previousthe incidence of pneumonia following cerebral surgery can increase the duration of hospital stays by up to 40%, compounded by the high morbidity associated with respiratory complications in older adults [42]. Furthermore, UTIs represent a common postoperative complication, exacerbated by factors such as the routine use of urinary catheters during and post-surgery. UTIs can extend even hospital stays by an average of 2.5 days [43]. This extension is attributed to the delays in mobilization and the additional treatment required to manage infection in an already immunocompromised patient group. multidisciplinary management of older surgical patients, integrating the expertise of surgeons, anesthetists, geriatricians, and other specialists and health care professionals. These roles might vary according to the phase and setting of care and the patient’s condition. hese findings underscore the importance of developing robust databases as well as implementing prehabilitation and Enhanced Recovery After Surgery (ERAS) protocols. Such measures are crucial not only for preventing complications but also for reducing the duration of hospital stays, which are associated with higher risks of non-surgical complications. Continuity of care is paramount for optimal treatment outcomes, necessitating early planning for anticipated needs, final care locations, and transition strategies for challenging cases, as suggested by Aceto et al. [44]

In this study, we observed a mortality rate of 3.9%, mainly due to pulmonary embolism and severe pneumonia, each accounting for two cases. This rate aligns closely with the 4% mortality rate reported in similar contexts [12, 35, 45]. In their study of 236 craniotomies for gliomas and brain metastases, Rabadán et al. (2007) also noted a 4% mortality rate in patients over 60 years old [46]. However, Stark et al. (2011) identified a much higher mortality rate of 18.2% in patients over 70 years old who underwent surgical removal of up to three brain metastases [26]. This variation in mortality rates across different studies underscores the need for national-level data analysis to gain a more comprehensive understanding of the outcomes of brain metastases surgery. In our department, we have implemented comprehensive review sessions for complex cases, involving our entire neurosurgical team. The goal of these sessions is to identify and address any potential gaps in patient care, thereby reducing the likelihood of AEs. This approach is part of our commitment to improving patient outcomes. Furthermore, we have adopted the Clavien-Dindo Classification system to standardize our documentation of AEs, enhancing both our internal processes and the comparability of our data with other neurosurgical centers [47, 48]. Morbidity and Mortality Conferences are a key aspect of our practice. These conferences provide a platform for our surgical teams to share experiences, critically analyze cases, and learn from past mistakes, thus preventing future occurrences of similar issues [49, 50]. This holistic approach not only enhances patient care but is also instrumental in shaping the next generation of neurosurgeons.

A substantial proportion of patients with solid tumors, estimated between 20 and 40%, are likely to develop brain metastases during the course of their disease and treatment [Bradley et al., 2021]. It is crucial to emphasize that survival rates among patients with BMs vary significantly due to a myriad of factors, including intra- and extracranial disease status, stability of systemic disease, cancer type, and the application of potential therapeutic strategies. In our current study, which focuses exclusively on older patients, we utilized the Diagnosis-Related Graded Prognostic Assessment (GPA) to predict overall survival, finding an average survival duration of 8 months. This aligns with existing literature indicating the variable incidence of BMs five years post-diagnosis of the primary tumor—16.3% for lung cancer, 5% for breast cancer, 9.8% for renal cancer, and 1.2% for colorectal cancer [51]. Recent reviews and meta-analyses have underscored the critical importance of classifying patients with BMs based on their extracranial disease status]. Findings from these studies suggest that patients with stable extracranial disease status typically exhibit a median overall survival of 17.9 months, whereas those with limited extracranial disease have a median survival of 8 months. Additionally, median overall survival (OS) in patients with BMs is influenced by various factors, including age, gender, KPS score, disease-free interval, status of extracranial metastases, number of metastases, and neurological status. Notably, favorable prognostic factors for survival include good baseline neurological status, a high KPS score (greater than 70), younger age, absence of extracranial metastases, and the presence of a solitary brain metastasis [52]. Reflecting on surgical outcomes, Sivasanker et al. reported in their retrospective analysis of 124 patients with BMs that factors such as female gender, age under 60 years, and intact neurological status at presentation were associated with improved median overall survival rates [53]. Specifically, they noted a significant difference in survival based on age; patients older than 60 years had a median survival of 6 months, compared to 13.2 months in younger patients.

### Limitations

One of the foremost strengths of our investigation is the utilization of a prospective database for analyzing adverse events (AEs), focusing specifically on a cohort of older patients with brain metastases (BM). Nevertheless, it is crucial to consider the limitations inherent in our study. A significant limitation is the follow-up period restricted to 30 days, which constrains our capacity to detect and appraise potential long-term complications. This timeframe was specifically chosen to assess perioperative risks and provide insights into short-term patient safety, which are crucial for guiding early postoperative care strategies. However, we recognize that a more extended follow-up period of 6–12 months would allow for a more thorough evaluation of long-term oncological outcomes, such as overall survival, local recurrence, and quality of life. Furthermore, despite thorough monitoring of each case, the possibility of misinterpreting certain events remains a concern. A factor that might have skewed our results is the initial KPS of the patients, which was generally favorable. This suggests that our study may have not fully accounted for older patients with a poor baseline health status. Furthermore, while it is important to document long-term survival rates, this is beyond the scope of this study. Another limitation of our study is the absence of a control group, such as a younger cohort or patients treated with SRS. The primary objective of this research was to focus on early postoperative outcomes and adverse events specific to elderly patients (≥ 65 years) undergoing surgical resection of brain metastases. Including a younger matched cohort or a group treated with SRS was beyond the intended scope of this study, as our main aim was to capture short-term perioperative complications in an often underrepresented population. While this could have enriched the findings, it does not detract from the relevance and significance of the results concerning treatment outcomes for the elderly. Another limitation of our study is the lack of detailed stratification based on the primary tumor types and molecular or genetic profiles. Given the focus of this research on short-term surgical outcomes and adverse events in elderly patients, our primary goal was to capture clinical and perioperative data that directly influence immediate postoperative management. While molecular profiling could provide additional insights into the oncological course and individualized treatment responses, this was beyond the scope and feasibility of the current study. Future research that incorporates genetic profiling and detailed tumor stratification would be valuable for understanding the broader implications of different tumor biology in patients undergoing surgical intervention for brain metastases. One of the limitations of our study is the lack of direct comparisons between the surgical outcomes of BM and other cranial pathologies, such as meningiomas, in elderly patients. Consequently, our findings should not be interpreted as definitive evidence that BM surgery bears fewer risks than surgeries for other types of cranial pathologies. We recognize this as an important gap in the current research, and it underscores the need for future studies specifically designed to compare these surgical outcomes. *While the duration of stay was longer (10 days)*,* our study did not specifically analyze data on the incidence of pulmonary or urinary tract infections and their direct impact on hospitalization length. This potential association remains speculative and should be explored further in future research.*

### Outlook

An important future direction for our research involves integrating comprehensive frailty assessments into the preoperative evaluation process of elderly patients undergoing surgery for brain metastases. While this study relied on the CCI and KPS to assess patient risk, future iterations will include more refined geriatric assessments, such as the Fried Frailty Index or the Clinical Frailty Scale, to capture the multidimensional aspects of frailty, including physical, cognitive, and social vulnerabilities. These tools will help refine patient selection and better predict outcomes, ensuring that surgical interventions are tailored to the individual needs of elderly patients, thereby optimizing both perioperative care and long-term recovery.

## Conclusions

Our findings affirm that resection of BM resulted in acceptable morbidity outcomes for elderly patients and may be associated with less adverse events than other cranial tumor surgeries. Nonetheless, the importance of vigilant pre– and postoperative monitoring cannot be overstated, particularly in light of the patients’ baseline health status which predisposes them to AEs after surgery. Establishing uniform protocols for reporting and analyzing AEs is therefore not just beneficial for clinical outcomes, but also for maintaining the integrity and trust in healthcare services provided to these patients.

## Data Availability

No datasets were generated or analysed during the current study.

## Code availability

The code supporting the findings of this study is available from the corresponding author upon reasonable request.
